# Paranormal Experience Profiles and Their Association With Variations in Executive Functions: A Latent Profile Analysis

**DOI:** 10.3389/fpsyg.2021.778312

**Published:** 2022-01-10

**Authors:** Kenneth Graham Drinkwater, Neil Dagnall, Andrew Denovan, Andrew Parker, Álex Escolà-Gascón

**Affiliations:** ^1^Department of Psychology, Manchester Metropolitan University, Manchester, United Kingdom; ^2^Adelphi Values Ltd., Bollington, United Kingdom; ^3^Blanquerna Foundation, Barcelona, Spain

**Keywords:** paranormal experience/belief, executive functions, latent profile analysis, self-report measures, metacognitive processes

## Abstract

This study investigated relationships between inter-class variations in paranormal experience and executive functions. A sample of 516 adults completed self-report measures assessing personal encounter-based paranormal occurrences (i.e., Experience, Practitioner Visiting, and Ability), executive functions (i.e., General Executive Function, Working and Everyday Memory, and Decision Making) together with Emotion Regulation and Belief in the Paranormal. Paranormal belief served as a measure of convergent validity for experience-based phenomena. Latent profile analysis (LPA) combined experience-based indices into four classes based on sample subpopulation scores. Multivariate analysis of variance (MANOVA) then examined interclass differences. Results revealed that breadth of paranormal experience was associated with higher levels of executive functioning difficulties for General Executive Function, Working Memory, Decision Making, and Belief in the Paranormal. On the Everyday Memory Questionnaire, scores differed on Attention Tracking (focus loss) and Factor 3 (visual reconstruction), but not Retrieval (distinct memory failure). In the case of the Emotion Regulation Scale, class scores varied on Expressive Suppression (control), however, no difference was evident on Cognitive Reappraisal (reframing). Overall, inter-class comparisons identified subtle differences in executive functions related to experience. Since the present study was exploratory, sampled only a limited subset of executive functions, and used subjective, self-report measures, further research is necessary to confirm these outcomes. This should employ objective tests and include a broader range of executive functions.

## Introduction

Drinkwater et al. (2021b) found that greater personal involvement with the paranormal (i.e., experience, visiting practitioners and self-professed ability) was associated with increased proneness to reality testing deficits, greater emotion-based reasoning, and higher paranormal belief. These results deepened conceptual understanding by demonstrating that nuanced variations in paranormal experience were related to subtle differences in cognitive-perceptual factors allied to subclinical delusion formation and thinking style. These outcomes concurred with those observed for paranormal belief (Irwin et al., 2012a,b; Williams et al., 2021).

Extending this work, the present paper investigated relationships between personal paranormal encounters and executive functions. Executive functions denote interrelated mental activities comprising top-down processes (Burgess and Simons, 2005; Diamond, 2013), specifically interference control (the ability to effectively select stimuli in accordance with set goals for further processing), working memory (short-term storage and manipulation of information), inhibition (self-control and resistance to acting impulsively), and cognitive flexibility (ability to think outside pre-established frameworks). These component processes play an integral role in everyday activities such as planning, recall, dual-tasking, and attentional focus (Diamond, 2013).

To date, only limited research has examined relationships between paranormal factors and executive functions. Moreover, published work has focused largely on beliefs, particularly religion and superstition. Although these domains are core facets of paranormality (Dagnall et al., 2010c), they represent only a narrow range of construct content. Furthermore, the fact these supernatural facets share important features constrains the generalizability of findings to other paranormal domains. Specifically, religious and superstitious beliefs derive from magical ideation, credence in forms of causation that are scientifically/conventionally invalid (Eckblad and Chapman, 1983), and persist in the absence of empirical support (Lindeman and Svedholm, 2012). In terms of executive functions, increased religiosity is linked to activity in frontal brain regions, explicitly the dorsolateral-pre-frontal cortex (Azari et al., 2001), and areas in the medial prefrontal cortex (Muramoto, 2004; Rim et al., 2019). Whereas superstition is concomitant with medial temporal lobe dysfunction, predominantly the hippocampus (Brugger et al., 1994).

Regarding religion, Schjoedt et al. (2013) contend that executive resource depletion plays a crucial role in sacred experiences. Specifically, three features (i.e., demand for the expressive suppression of emotion, exposure to goal demoted and causally opaque actions, and presence of a charismatic authority) limit the capacity for individual processing of religious events, and concurrently increase susceptibility to authoritative narratives. In support of the cognitive resource depletion model (Schjoedt et al., 2013), studies have shown that reductions in available resources (decreased working memory capacity) are related to increased paranormal belief (Richards et al., 2014). Additionally, Richards et al. (2014) found that paranormal belief was positively associated with inattentional blindness. This is the inability to register an unexpected visual stimulus or event when attention is engaged on another task (Mack, 2003). This is an important observation, since inattentional blindness is partially dependent upon reduced top-down processing (executive control) (Hannon and Richards, 2010).

Lack of executive control can also result in increased susceptibility to proactive interference and the tendency to perceive random coincidences as causally related (Rominger et al., 2011). The inclination to see patterns within meaningless stimuli is a significant prognosticator of higher levels of paranormal belief (Dagnall et al., 2007, 2014, 2016a,b; Escolà-Gascón, 2020). Correspondingly, the ability to overcome interference plays a central role in working memory (Lustig et al., 2001; Rominger et al., 2019).

Other biases related to paranormal belief (i.e., false perceived agency, Riekki et al., 2014; and faces in noise, Riekki et al., 2013) are explicable in terms executive functioning. Particularly, they denote failures to actively inhibit or exercise sufficient control over the influence of top-down signals on the processing of sensory data. Neuroimaging studies support this supposition. For example, Lindeman et al. (2013) found that paranormal believers reported seeing more illusory “signs” (indications or symbolic hints) in neutral pictures (e.g., a brick wall) when imagining personal scenarios. This tendency aligns with lower activity in the inferior frontal gyrus, which is activate during cognitive inhibition (e.g., Aron et al., 2004, 2014).

Further research reports links between variations in dopaminergic activity, paranormal belief, and executive functions. These relationships are potentially attributable to the fact that the pre-frontal region receives dopaminergic inputs from the midbrain and expresses a variety of dopamine receptor subtypes (Logue and Gould, 2014; Ott and Nieder, 2019). Pertinent to the present paper, Krummenacher et al. (2010) reported that alterations in dopaminergic activity altered cognitive processing in skeptics. Subsequently, they performed more like paranormal believers on a perceptual task requiring the detection of meaningful patterns amidst noise. Krummenacher et al. (2010) concluded that this was imputable to dopamine reducing perceptual sensitivity (the signal to noise ratio). This is consistent with the observation that greater internal noise (compared to signal) can produce perceptual aberrations, generate delusions, and enhance superstitious beliefs (Shaner, 1999). These outcomes are commensurate with the view that executive processes play an integral role in error monitoring (van Elk and Aleman, 2017).

Extending previous research on cognitive-perceptual personality factors (Drinkwater et al., 2021a,b), this paper conducted an exploratory investigation to determine whether differences in the breadth and intensity of paranormal experiences were associated with variations in neuropsychological measures related to self-reported executive functions (i.e., memory and decision making). In addition, the ability to control or regulate emotional responses was assessed. Emotion regulation was included as past work has shown associations between executive-control functions and emotions plays a role in anomalous experiences (Schjoedt et al., 2013). That is, similar frontal regions that form the neural basis of executive functioning also influence emotional expressive control and appraisal (e.g., Zelazo and Cunningham, 2007; Ochsner et al., 2009; Schmeichel and Tang, 2015). These regions have additionally been theorized to be a factor underpinning anomalous experience such as religion (Grafman et al., 2020). Accordingly, this study explored how emotional regulation functions in the context of paranormal belief and ability.

Finally, the level of paranormal belief served as a measure of convergent validity for experience-based phenomena. This was necessary because experience-based indices (i.e., Experience, Practitioner Visiting, and Ability) were amalgamated to form “novel” composite measures. Using Cohen (1988) effect size guidelines, consistent with preceding research, moderate-large positive correlations should be observed between belief and experience-based measures. Furthermore, Experience, Practitioner Visiting, and Ability should be positively related to belief but make distinct, unique contributions.

Consistent with Drinkwater et al. (2021b), the present study used latent profile analysis (LPA) to combine personal encounter-based phenomena (Experience, Practitioner Visiting, and Ability). LPA extends research in several ways. Specifically, it identifies important, nuanced variations in individual supernatural histories/perceptions, assesses a broader range of factors than traditional measures (i.e., subjective paranormal experience), and acknowledges that experience of the paranormal is heterogeneous (see Drinkwater et al., 2021b). Furthermore, the application of LPA to belief has provided important conceptual insights (see Denovan et al., 2018).

## Methods

### Design

Data collection occurred at one point in time. A frequent criticism of this cross-sectional approach is vulnerability to common method variance (CMV) (Spector, 2019). To counter CMV, the present investigation implemented procedural remedies (Krishnaveni and Deepa, 2013). Explicitly, instructions to respondents created psychological distance between scales by emphasizing the unique nature of each construct (Podsakoff et al., 2003). In addition, to lessen possible social desirability and evaluation apprehension effects, directions informed respondents that there were no correct answers.

### Respondents

The sample comprised 516 respondents, mean age (*M*) = 40.17 years, SD = 16.85, range 18–87. There were 225 males (44%), *M* = 40.96 years, SD = 17.89, range 18–87; and 290 females (56%), *M* = 39.62 years, SD = 16.00, range 18–75. One participant failed to specify gender. Data screening revealed that skewness and kurtosis values were acceptable; within −2.0 to + 2.0 (Byrne, 2013). Respondent recruitment was *via* Bilendi, an established online provider of quality, representative samples (Salak et al., 2021). Generally, panel data is diverse, far reaching, and comparable with traditional methods (Kees et al., 2017). The researchers requested United Kingdom-based respondents aged 18 years and over.

### Measures

#### Experiential Paranormal Factors

Personal encounter-based phenomena comprised Experience, Practitioner Visiting, and Ability (see Dagnall et al., 2016c; Drinkwater et al., 2020, 2021a,b).

##### Experience

Items established whether respondents had directly encountered commonly reported, core receptive paranormal phenomena (see Drinkwater et al., 2013; Dagnall et al., 2016c; Drinkwater et al., 2017a,^2018^). Explicitly, psi (i.e., mental abilities/powers) and life after death (i.e., communication with the deceased). Experiences included psychic occurrence, mediumship, spiritualism, telepathy, precognition, premonition, and remote viewing. The provision of precise conceptual based definitions ensured that respondents were aware of the nature of each phenomenon. For example, “Spiritualists provide information regarding the transition of the human spirit from the physical body to the afterlife. In the context of this definition, have you ever personally experienced spiritualism?” Totaling experiences produced a score ranging from 0 (no experience) to 8 (experienced all phenomena). This is a well-established, reliable method for assessing self-reported paranormal experiences (see Dagnall et al., 2016c; Drinkwater et al., 2020, 2021a,b).

##### Practitioner Visiting

Questions enquired whether respondents had visited paranormal practitioners in areas allied to psi and life after death. Classifications reflected the foremost industries (i.e., Mediums, Psychics, Spiritualists, and Fortune-Tellers). Respondents indicated whether they had visited each practitioner type using a dichotomous format (yes vs. no). This scale has demonstrated satisfactory internal reliability (Drinkwater et al., 2021b). Scores thus ranged from 0 to 4, with higher scores indicating greater interaction with paranormal practitioners.

##### Self-Perceived Ability

Items asked respondents whether they believed that they possessed paranormal ability in each of the core phenomena (i.e., mediumship, psychic, spiritualism, and fortune telling). For instance, “To what extent (in percentage terms, 0–100) do you believe that you possess psychic abilities?” This method has excellent internal consistency (Drinkwater et al., 2021b). A further item asked whether respondents were paranormal practitioners (yes vs. no). These measures allowed the researchers to categorize respondents as no ability, self-professed ability (moderate), and practitioners (high). Previous research has established the validity of these groupings and that they differ in intensity of personal ascription of level of ability (see Drinkwater et al., 2021a).

#### Measures of Executive Function

Self-report measures assessed executive functioning including General Executive Functions, Memory, and Decision Making.

##### General Executive Functions (Webexec)

The Webexec (Buchanan et al., 2010) is a measure of executive functioning problems designed for internet-mediated research. The scale contains six items assessing difficulties in maintaining focus, concentration, multitasking, sustaining a train of thought, completing tasks, and acting impulsively. Items appear as questions (e.g., “Do you find it difficult to keep your attention on a particular task?”) and participants respond *via* a four-point Likert scale (1 = no and 4 = many problems). Summation of item scores produces a total ranging from 6 to 24, with higher scores indicating greater executive functioning problems. The Webexec has demonstrated construct and convergent validity, and satisfactory internal reliability (Buchanan et al., 2010). In this study, good alpha reliability existed, α = 0.89.

##### The Everyday Memory Questionnaire–Revised

The 13-items Everyday Memory Questionnaire–Revised (EMQ-R) (Royle and Lincoln, 2008) assesses everyday memory-related behaviors (e.g., “Completely forgetting to do things you said you would do, and things you planned to do.”). Participants respond by providing an estimate of how many times item content has occurred over the previous month (0 = once or less, 1 = more than once a month but less than once a week, 2 = about once a week, 3 = more than once a week but less than once a day, 4 = once or more in a day). The scale encompasses three subscales measuring Retrieval (i.e., memory failure), Attention Tracking (focus loss), and Factor 3 (visual reconstruction). Previous studies indicate that the EMQ-R has sound psychometric properties (Royle and Lincoln, 2008). These subscales demonstrated good to satisfactory reliability; Retrieval (α = 0.92), Attention Tracking (α = 0.87), and Factor 3 (α = 0.73).

##### The Working Memory Questionnaire

The Working Memory Questionnaire (WMQ) (Vallat-Azouvi et al., 2012) comprises 30 items investigating three domains of working memory: short-term storage (the ability to retain information for a brief period), attention (i.e., distractibility, mental slowness, mental fatigue, and dual-task processing), and executive function (i.e., decision making, planning, and shifting). Items appear as questions (e.g., “Do you have difficulty remembering what you have read?”) and participants respond *via* a five-point Likert scale, ranging from 0 (“no problem at all”) to 4 (“very severe problem in everyday life”). Higher scores represent greater working memory difficulties. The WMQ is a robust, psychometrically validated scale (Vallat-Azouvi et al., 2012). Excellent reliability existed in this study, α = 0.97.

##### Decision Making Questionnaire

The present study used the Control and Instinctiveness subscales of the Decision Making Questionnaire (DMQ) (French et al., 1993) to assess decision making efficacy. These dimensions evaluate impulsiveness (hastiness), which is a key component of executive functioning. Items appear as questions and participants respond, using a six-point Likert scale ranging from 1 (“Very infrequently or never”) to 6 (“Very frequently or always”). The scale has been psychometrically validated (French et al., 1993) and used in published studies (Douse and McManus, 1993; Kumar and Gupta, 2017). For the current study, the composite measure demonstrated satisfactory reliability, α = 0.73.

#### Other Measures

Additional measures assessed self-reported ability to control emotional responses (Emotional Regulation) and Paranormal Belief.

##### Emotion Regulation Questionnaire

The Emotion Regulation Questionnaire (ERQ) (Gross and John, 2003) includes 10 items that evaluate dispositional emotion regulation strategies. Specifically, expressive suppression (i.e., “I control my emotions by not expressing them”) and cognitive reappraisal (i.e., “When I want to feel more positive emotions, I change the way I’m thinking about the situation”). The scale presents items as statements and participants respond by completing a seven-point Likert scale (1 = strongly disagree, 7 = strongly agree). The ERQ has established psychometric integrity (Gross and John, 2003). In the present study, internal reliability was adequate (expressive suppression, α = 0.60; cognitive reappraisal, α = 0.60).

##### Belief in the Paranormal (Revised Paranormal Belief Scale)

The Revised Paranormal Belief Scale (RPBS) (Tobacyk, 2004) is a 26-item measure of belief in the paranormal. Items appear as statements (e.g., “I believe in God”) and participants provide responses using a seven-point Likert scale, ranging from 0 (strongly disagree) to 6 (strongly agree) (see Lange et al., 2000). Total scores range from 0 to 156, with higher scores indicating belief in the paranormal. The RPBS is a widely used, psychometrically satisfactory measure (Drinkwater et al., 2017b). Internal reliability in this study was excellent, α = 0.96.

### Procedure

Prospective respondents accessed information by clicking on a web link. After reading the participant information sheet, only respondents providing informed consent progressed. Subsequent instructions then directed respondents to read and complete all items, provide truthful answers, and progress through sections at their own pace. The survey contained subsections comprising demographic characteristics (i.e., age and preferred gender), paranormal experiences, measures of executive functioning, and paranormal belief. To prevent potential order effects, sections and scales rotated across respondents. On completion of the study, respondents were debriefed.

### Ethics Statement

Ethical approval was provided by the Manchester Metropolitan University, Faculty of Health, Psychology and Social Care Ethics Committee (October 2018; Project ID, 954).

## Results

### Analytical Strategy

Initially analysis examined descriptive relationships. Then, using Mplus version 7 (Muthén and Muthén, 2012), group membership was established by combining personal encounter-based paranormal measures (Experience, Practitioner Visiting, and Ability). Model fit evaluated a 1-class model, followed by iterations with increasing latent profiles. A range of indices determined the number of latent profiles (classes): Akaike Information Criterion (AIC; Akaike, 1987), Bayesian Information Criterion (BIC; Schwarz, 1978), sample-size adjusted BIC (ssaBIC; Sclove, 1987), Lo-Mendell-Rubin-adjusted likelihood ratio test (LMR-A-LRT; Lo et al., 2001), and a measure of entropy (Ramaswamy et al., 1993). Lower values for AIC, BIC, and ssaBIC indicate greater fit in addition to consideration of LMR-A-LRT and entropy. LMR-A-LRT tested significance of fit (*via* a *p* value). For entropy, values above 0.8 specify sound separation of profiles relative to data (Ramaswamy et al., 1993). Finally, analysis using SPSS26 explored class differences on study measures.

### Descriptive Statistics

Data screening identified seven z-scores marginally greater than 3.25. Prior to analysis, the researchers transformed these to the next highest score (Tabachnick and Fidell, 2001). Correlations (Table 1) revealed personal encounter-based indices (Experience, Practitioner Visiting, and Ability) correlated positively. Paranormal Belief, General Executive Functioning, Working Memory, Decision Making, Emotion Regulation correlated positively, as did Emotion Regulation and Everyday Memory subscales. Several associations were small (*r* = 0.10–0.30) (Cohen, 1992). However, using Gignac and Szodorai’s (2016) guidelines these signify meaningful relationships (i.e., small, *r* = 0.11; medium, *r* = 0.19, and large, *r* = 0.29).

**TABLE 1:** Descriptive statistics and correlations among variables.

Variable	Mean	SD	1	2	3	4	5	6	7	8	9	10	11	12
1. Paranormal Experience				0.73**	0.76**	0.67**	0.21**	0.21**	0.25**	0.04	0.14*	0.12*	0.11*	0.26**
2. Paranormal Practitioner Visiting					0.59**	0.57**	0.17**	0.16**	0.25**	0.06	0.14*	0.13*	0.13*	0.23**
3. Paranormal Ability						0.63**	0.24**	0.31**	0.14*	0.16**	0.25**	0.23**	0.03	0.17**
4. Paranormal Belief	71.20	34.44					0.30**	0.32**	0.11*	0.21**	0.32**	0.24**	0.12*	0.21**
5. Executive Function	12.15	4.38						0.68**	−0.34**	0.62**	0.61**	0.45**	−0.05	−0.03
6. Working Memory	33.93	26.14							−0.39**	0.75**	0.80**	0.71**	0.04	0.02
7. Decision Making	26.50	5.71								−0.36**	−0.29**	−0.23**	0.29**	0.34**
8. Retrieval	8.54	7.19									0.84**	0.72**	−0.02	−0.02
9. Attention	3.88	4.10										0.78**	0.02	−0.01
10. Factor 3	1.49	2.03											0.06	0.05
11. Cognitive reappraisal	26.94	5.88												0.76**
12. Expressive suppression	18.48	4.21												

* indicates p < 0.05; ** indicates p < 0.001.

### Latent Profile Analysis

Assessment of 1-class and 2-class models revealed superior fit for the 2-class model (Table 2), with lower AIC, BIC, ssaBIC, and a significant LMR-A-LRT. Comparison of 2-class and 3-class models found the 3-class solution superior.

**TABLE 2:** Fit of latent profile models.

Model	AIC	BIC	ssaBIC	LMR-A	LMR-A *p* value	Entropy
1-class	5,555.46	5,580.94	5,561.90			
2-class	4,531.91	4,574.37	4,542.63	991.85	<0.001	0.78
3-class	3,784.66	3,971.49	3,831.83	129.35	<0.001	0.82
4-class	3,789.98	4,040.50	3,853.22	24.42	0.03	0.94
5-class	3,801.21	4,115.42	3,880.53	18.57	0.60	0.85

*AIC, Akaike Information Criterion; BIC, Bayesian Information Criterion; ssaBIC, sample-size adjusted BIC; LMR-A, Lo-Mendell-Rubin-adjusted likelihood ratio test.*

**FIGURE 1:**
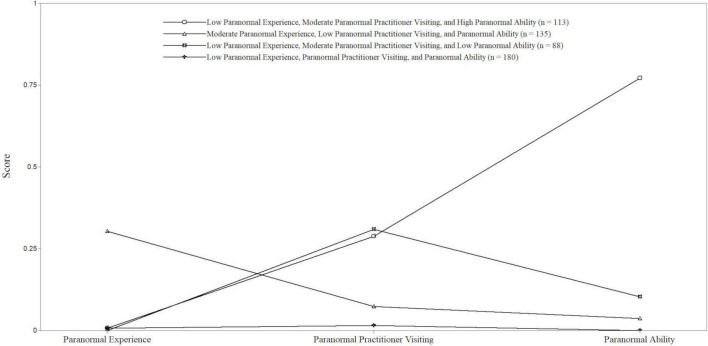
Latent profiles in relation to Paranormal Experience, Paranormal Practitioner Visiting, and Paranormal Ability (scores range from 0 to 1 since they are depicted as probabilities/percentages).

The 4-class solution was selected as the final model. Specifically, although greater AIC, BIC and ssaBIC existed, it possessed a considerably lower (and significant) LMR-A-LRT value vs. the 3-class model alongside higher entropy (0.94 vs. 0.82). The three- and four-class models were, nonetheless, examined closely, as they both could have legitimate arguments for being selected. Empirical factors, such as greater separation among profiles, indicated a more defensible basis for selecting the 4-class solution. Finally, the 5-class model failed to improve the model (see Figure 1). Average latent class probabilities for latent profile membership were 0.92 for Class 1 (Low Experience, Moderate Visiting, and High Ability), 0.86 for Class 2 (Moderate Experience, Low Visiting, and Ability), 0.85 for Class 3 (Low Experience, Moderate Visiting, and Low Ability), and 0.89 for Class 4 (Low Experience, Visiting, and Ability), indicating good discrimination.

### Association of Latent Profiles With Neuropsychological Measures

Multivariate analysis of variance (MANOVA) assessed latent profiles on Paranormal Belief, General Executive Function, Working Memory, and Decision Making (Table 3). Analysis revealed a significant main effect of group, Pillai’s trace = 0.57, *F*(12, 1,533) = 30.40, *p* < 0.001, η^2^ = 0.19 (large effect size). All outcomes differed significantly.

**TABLE 3:** Latent profile effects on paranormal belief, executive function, working memory, and decision making.

		Dependent variable					
	Paranormal Belief	Executive Function	Working Memory	Decision Making			
				
	ANOVA					MANOVA	
	*F *^df^** (*Sig.*; η^2^)	*F *^df^** (*Sig.*; η^2^)	*F *^df^** (*Sig.*; η^2^)	*F *^df^** (*Sig.*; η^2^)	Pillai	*F *^df^** (*Sig.*)	η^2^
Variable Group	152.64^3,512^ (<0.001; 0.47)	21.57^3,512^ (<0.001; 0.11)	17.27^3,512^ (<0.001; 0.09)	17.29^3,512^ (<0.001; 0.09)	0.57	30.40^15,^ ^1,530^ (<0.001)	0.19

	Pairwise comparisons (mean differences) between classes						
Contrast	Mean diff. (*Sig.*)	Mean diff. (*Sig.*)	Mean diff. (*Sig.*)	Mean diff. (*Sig.*)			

Class 1 v. 2	41.25 (<0.001)	0.92 (0.47)	6.09 (0.33)	4.43 (<0.001)			
Class 1 v. 3	12.96 (0.002)	−1.89 (0.008)	−7.16 (0.26)	4.40 (<0.001)			
Class 1 v. 4	58.92 (<0.001)	2.28 (<0.001)	14.56 (<0.001)	3.77 (<0.001)			
Class 2 v. 3	−28.28 (<0.001)	−2.82 (<0.001)	−13.26 (<0.001)	−0.03 (1.00)			
Class 2 v. 4	17.67 (<0.001)	1.35 (0.025)	8.46 (0.018)	−0.65 (1.00)			
Class 3 v. 4	45.96 (<0.001)	4.18 (<0.001)	21.72 (<0.001)	0.63 (1.00)			

*Class 1, Low Experience, Moderate Visiting and High Ability; Class 2, Moderate Experience, Low Visiting, and Ability; Class 3, Low Experience, Moderate Visiting and Low Ability; Class 4, Low Experience, Visiting, and Ability.*

*Post hoc* Bonferroni comparisons (Table 3) revealed that Class 1 demonstrated highest Belief in the Paranormal and Decision Making. Class 3 reported highest Executive Function and Working Memory disruption. Class 4 had significantly lower scores on all outcomes, with the exceptions of Class 2 and Class 3 on Decision Making.

### Emotion Regulation and Everyday Memory Subscale Analysis

Further MANOVA analysis examined differences on Emotion Regulation (Cognitive Reappraisal and Expressive Suppression) and Everyday Memory (Retrieval, Attention Tracking, and Factor 3) subscales (Table 4). A significant main effect occurred, Pillai’s trace = 0.16, *F*(15, 1,530) = 5.88, *p* < 0.001, η^2^ = 0.06 (medium effect size). Significant group effects existed for all variables but Cognitive Reappraisal. *Post hoc* Bonferroni comparisons (Table 4) revealed that Class 4 scored significantly lower than Class 1 on Attention Tracking Factor 3, and Expressive Suppression, and lower than Class 3 on Retrieval, Attention Tracking, and Factor 3. A less consistent pattern existed for remaining profiles. Specifically, Class 1 scored highest on Expressive Suppression, and Class 3 scored highest on Retrieval, Attention Tracking, and Factor 3.

**TABLE 4:** Latent profile effects on emotion regulation and everyday memory subscales.

	Everyday Memory			Emotion Regulation				
	Retrieval	Attention Tracking	Factor 3	Cognitive Reappraisal	Expressive Suppression			
	**ANOVA**						**MANOVA**	
	*F *^df^** (*Sig.*; η^2^)	*F *^df^** (*Sig.*; η^2^)	*F *^df^** (*Sig.*; η^2^)	*F *^df^** (*Sig.*; η^2^)	*F *^df^** (*Sig.*; η^2^)	Pillai	*F *^df^** (*Sig.*)	η^2^

Variable Group	7.44^3,512^ (<0.001; 0.04)	9.68^3,512^ (<0.001; 0.05)	6.64^3,512^ (<0.001; 0.04)	2.28^3,512^ (0.079; 0.01)	11.50^3,512^ (<0.001; 0.06)	0.16	5.88^15,^ ^1,530^ (<0.001)	0.06

	Pairwise comparisons (mean differences) between classes							
Contrast	Mean diff. (*Sig.*)	Mean diff. (*Sig.*)	Mean diff. (*Sig.*)	Mean diff. (*Sig.*)	Mean diff. (*Sig.*)			

Class 1 v. 2	−0.23 (1.00)	0.70 (0.996)	0.27 (1.00)	1.27 (0.536)	2.31 (<0.001)			
Class 1 v. 3	−3.22 (0.008)	−0.96 (0.540)	−0.47 (0.583)	1.58 (0.343)	1.67 (0.025)			
Class 1 v. 4	1.09 (1.00)	1.67 (0.003)	0.64 (0.048)	1.76 (0.074)	2.78 (<0.001)			
Class 2 v. 3	−2.98 (0.013)	−1.67 (0.014)	−0.74 (0.042)	0.31 (1.00)	−0.64 (1.00)			
Class 2 v. 4	1.33 (0.587)	0.96 (0.208)	0.37 (0.632)	0.49 (1.00)	0.47 (1.00)			
Class 3 v. 4	4.32 (<0.001)	2.64 (<0.001)	1.11 (<0.001)	0.17 (1.00)	1.11 (0.220)			

*Class 1, Low Experience, Moderate Visiting and High Ability; Class 2, Moderate Experience, Low Visiting, and Ability; Class 3, Low Experience, Moderate Visiting and Low Ability; Class 4, Low Experience, Visiting, and Ability.*

## Discussion

Based on differences in Experience, Visiting, and Ability, latent profile analysis (LPA) identified four subpopulations. These represented variations in individual supernatural histories and reflected discrete and complex interactions between experiential indices. In this context, consistent with Drinkwater et al. (2021b), LPA advanced experience-based research by acknowledging the broad and heterogeneous empirical basis of supernatural phenomena. Additionally, LPA recognized importance intra-class communalities and disparities.

From a methodological perspective, creation of classes based on multiple experience factors is an important development because preceding work has typically relied upon single measures. For instance, theorists have placed an overreliance on “subjective paranormal experiences” (SPEs) (e.g., Palmer and Neppe, 2004; Dagnall et al., 2016c). Although consideration of SPEs has provided valuable insights into individual perceptions of paranormality, the construct is limited as it references only one encounter type. Precisely, willingness to attribute supernatural causation to a specific event or occurrence. Despite this, since SPE inclusion facilitates direct comparisons with preceding findings, ensuing work should continue to use SPEs as a component within multiple measures.

The addition of Visiting and Ability alongside Experience ensured that the present study sampled a range of personal paranormal occurrences. In this context, the application of LPA enabled amalgamation of discrete but related factors to form conceptually meaningful classes. Consideration of zero-order correlations supported this approach. Explicitly, although intra-experiential measure associations were high, there remained a large proportion of unexplained variance. This, as predicted, indicated that while Experience, Visiting, and Ability overlap, each factor makes a unique contribution.

In comparison to Drinkwater et al. (2021b), the observed relationships in the present paper, both within and between experiential measures and paranormal belief, were stronger. Given sample equivalence, this suggests that scores derived from multiple indices of experience may be subject to variation. Further research should investigate this. Nonetheless, findings supported the notion that experiential paranormal factors and belief are inherently interconnected and reciprocally reinforcing (Dagnall et al., 2015, 2020; van Elk, 2017).

Class comparisons found significant differences across measures of executive function (General, Everyday and Working Memory, Emotion Regulation, and Decision Making) and Belief in the Paranormal. These were consistent for the highest (Class 1, Low Experience, Moderate Visiting and High Ability) vs. least experience (Class 4, Low Experience, Visiting, and Ability) groups. Class 1 (vs. Class 4) membership was associated with greater reported executive functioning difficulties. Analysis of Everyday Memory and Emotion Regulation scales revealed commensurate variations in performance. Everyday Memory, Class 4 (vs. Class 1) reported higher incidence of Attentional Tracking (sustained focus) and Factor 3 (visual reconstruction) difficulties; Retrieval (distinct memory failure) scores did not differ significantly. Emotion Regulation, Class 4 scored higher on Expressive Suppression (control).

Intermediate group comparisons (Class 2, Moderate Experience, Low Visiting, and Ability vs. Class 3, Low Experience, Moderate Practitioner Visiting and Low Ability) found that Class 3 reported higher levels of disruption on General Executive Function, Working Memory, Everyday Memory and Paranormal Belief. No differences were evident on Decision Making and Emotion Regulation. Finally, contrasts between the high (Class 1) and low (Class 4) and intermediate (Class 2 and Class 3) groupings identified further subtle variations in executive functioning. Collectively, outcomes indicated a relationship between range of paranormal encounters and self-reported executive functioning; greater experience was associated with higher levels of perceived disruption. This outcome aligned with Drinkwater et al. (2021b), who reported experience related differences on cognitive-perceptual factors allied to thinking style and delusion formation (reasons for these differences are discussed within section “Limitations and Future Research”).

### Limitations and Future Research

Although the present paper was exploratory and intended only to indicate potential pathways for subsequent projects, it is important to acknowledge limitations. Firstly, selection of experiences was limited to core receptive phenomena. Future studies should include additional commonly reported occurrences such as superstitious behaviors. Secondly, paranormal experience has a broader base than that assessed by Experience, Visiting, and Ability. Hence, ensuing research should include additional indices. For example, engagement with paranormal media (documentaries, reality programs, etc.), group affiliation (i.e., clubs), and background (family/friends). This could also examine which combination of factors are most representative of paranormal experience and most likely to predict executive functioning variations.

Thirdly, the researchers used self-report measures to generate data. The degree to which subjective, extemporaneous evaluations accurately reflect higher-order processes is questionable due to propensity to interpretative bias. Moreover, individuals may not possess access to complex cognitive-perceptual processes (as opposed to metacognitive evaluations or implicit *a priori* theories of cognitive functioning) (Nisbett and Wilson, 1977; Dagnall et al., 2008a; Chan et al., 2015). A related concern is that increased disruption scores may represent differences in abstractive focus (i.e., preoccupation with internal processes) rather than actual decrements in executive functions. This supposition is based on the observation that experience of and belief in the paranormal correlate positively with proneness to reality testing deficits (Dagnall et al., 2018), and a range of individual differences variables associated with odd and unusual cognitions, perceptions, and sensations (i.e., schizotypy and transliminality; Dagnall et al., 2010b). These relationships, in part, explicate why paranormal attributions are typically less structured, more productive/fluid, and involve heightened stimulus sensitivity.

Additionally, the notion of preoccupation with internal processes is consistent with the finding that paranormal believers prefer intuitive, experiential-based evidence (Irwin and Young, 2002; Dagnall et al., 2010a; Drinkwater et al., 2012). Tentatively, this concurs with the ability to main external attention and avoid unnecessary disruption. This may explain why believers are more inclined to metacognitive errors such as false memories (French, 2003; Dagnall et al., 2008b). Thus, to ensure that higher levels of reported executive function disruption are not simply artifacts of cognitive style/personality, succeeding investigations should attempt to replicate current outcomes using objective neuropsychological tests (see Buchanan, 2016). These should include fine-grained analysis of the subset of executive functions included in this paper (i.e., general executive function, working memory, decision making, and emotion regulation), and additional elements such as attentional control and cognitive inhibition.

Finally, LPA is statistically driven rather than theoretically derived. Hence, it can generate profiles that lack conceptual coherence. In the present study, classes represented meaningful variations in experience, which corresponded to differences in paranormal belief. Subsequent work should iteratively seek to develop standardized experience measures. Part of this process requires cross-validation methods, such as double cross-validation (Collins et al., 1994) or progressive elaboration (Donovan and Chung, 2015). These are essential to prevent LPA misspecification. In this context, cross-validation methods objectively evaluate model fit and assess class stability.

## Data Availability Statement

The raw data supporting the conclusions of this article will be made available by the authors, without undue reservation.

## Ethics Statement

The studies involving human participants were reviewed and approved by the Manchester Metropolitan University, Faculty of Health, Psychology and Social Care Ethics Committee (October 2018; Project ID, 954). The patients/participants provided their written informed consent to participate in this study.

## Author Contributions

KD and ND designed the study. KD researched and collated measures and organized data collection. AP appraised measures and content on executive functions. AD conducted all analyses, which were checked by ÁE-G. ND summarized findings and synthesized content for all sections. KD, AD, AP, and ÁE-G edited the final manuscript and prepared the draft for submission. All authors contributed to the article and approved the submitted version.

## Conflict of Interest

AD is employed by the company Adelphi Values Ltd. The remaining authors declare that the research was conducted in the absence of any commercial or financial relationships that could be construed as a potential conflict of interest.

## Publisher’s Note

All claims expressed in this article are solely those of the authors and do not necessarily represent those of their affiliated organizations, or those of the publisher, the editors and the reviewers. Any product that may be evaluated in this article, or claim that may be made by its manufacturer, is not guaranteed or endorsed by the publisher.
